# COVID-19 in Solid Organ Transplant Recipient: Exploring Cumulative Incidence, Seroprevalence and Risk Factors for Disease Severity

**DOI:** 10.3390/biology10121349

**Published:** 2021-12-18

**Authors:** Rossana Caldara, Paola Maffi, Sabrina Costa, Elena Bazzigaluppi, Cristina Brigatti, Vito Lampasona, Paola Magistretti, Fabio Manenti, Ilaria Marzinotto, Silvia Pellegrini, Marina Scavini, Antonio Secchi, Lorenzo Piemonti

**Affiliations:** 1 Clinical Transplant Unit, IRCCS Ospedale San Raffaele, 20132 Milan, Italy; caldara.rossana@hsr.it (R.C.); maffi.paola@hsr.it (P.M.); secchi.antonio@hsr.it (A.S.); 2 Faculty of Medicine and Surgery, Vita-Salute San Raffaele University, 20132 Milan, Italy; 3 Diabetes Research Institute, IRCCS Ospedale San Raffaele, 20132 Milan, Italy; costa.sabrina@hsr.it (S.C.); bazzigaluppi.elena@hsr.it (E.B.); brigatti.cristina@hsr.it (C.B.); lampasona.vito@hsr.it (V.L.); magistretti.paola@hsr.it (P.M.); manenti.fabio@hsr.it (F.M.); marzinotto.ilaria@hsr.it (I.M.); pellegrini.silvia@hsr.it (S.P.); scavini.marina@hsr.it (M.S.)

**Keywords:** COVID-19, pancreas transplantation, islet transplantation, seroprevalence, SARS-CoV-2

## Abstract

**Simple Summary:**

It is still uncertain whether recipients of solid organ transplant (SOT) are at increased risk of SARS-CoV-2 infection and/or poor outcomes due to COVID-19 in comparison to the general population. In this study, we report the cumulative incidence and outcomes of SARS-CoV-2 infection in a cohort of 291 SOT recipients. The COVID-19 cumulative incidence in SOT recipients resulted slightly higher compared to that of age-matched population during the study period. Moreover, the SARS-CoV-2 antibody frequency was around 2.6-fold higher than the incidence of cases who tested positive for SARS-CoV-2 RT-PCR, suggesting that the number of SOT recipients infected with SARS-CoV-2 is likely higher than described. In symptomatic recipients, kidney transplant was associated with a higher risk of developing moderate/critical disease, while common risk factors, including age and comorbidities, resulted less relevant for COVID-19 severity. Due to the high estimated crude mortality, symptomatic SOT recipients should be considered at high risk in case of SARS-CoV-2 infection.

**Abstract:**

Background: Solid organ transplant (SOT) recipients may be at increased risk for severe disease and mortality from COVID-19 because of immunosuppression and prolonged end-stage organ disease. As a transplant center serving a diverse patient population, we report the cumulative incidence and outcomes of SARS-CoV-2 infection in our cohort of SOT recipients. Methods: We prospectively included in this observational study SOT recipients with a functioning kidney (*n* = 201), pancreas ± kidney (*n* = 66) or islet transplant (*n* = 24), attending outpatient regular follow-up at the San Raffaele Hospital from February 2020 to April 2021. Antibodies to SARS-CoV-2 were tested in all patients by a luciferase immunoprecipitation system assay. Results: Of the 291 SOT recipients, 30 (10.3%) tested positive for SARS-CoV-2 during the study period and prevalence was not different among different transplants. The SARS-CoV-2 antibody frequency was around 2.6-fold higher than the incidence of cases who tested positive for SARS-CoV-2 RT-PCR. As for the WHO COVID-19 severity classification, 19 (63.3%) SOT recipients were mild, nine (30%) were moderate, and two were critical and died yielding a crude mortality rate in our patient population of 6.7%. Kidney transplant (OR 12.9 (1.1–150) *p* = 0.041) was associated with an increased risk for moderate/critical disease, while statin therapy (OR 0.116 (0.015–0.926) *p* = 0.042) and pancreas/islet transplant (OR 0.077 (0.007–0.906) *p* = 0.041) were protective. Conclusions: The incidence of SARS-CoV-2 infection in SOT recipients may be higher than previously described. Due to the relative high crude mortality, symptomatic SOT recipients must be considered at high risk in case of SARS-CoV-2 infection.

## 1. Introduction

On 21 February 2020 the first diagnosed case of COVID-19 was confirmed in Lombardy, a region of Northern Italy. On 8 March 2020, the entire Lombardy region went into lockdown with the rest of the country, and it quickly became a hotspot on the wold map of the COVID-19 pandemic. On 3 June 2020, free movement within the entire national territory was restored, de facto signaling the end of the SARS-CoV-2 associated disease first wave. Starting in July 2020, Italy witnessed a new progressive rise in COVID-19 cases, resulting in a second wave in November 2020 and a third wave in March 2021, as a result of the spreading of the Delta variant of SARS-CoV-2. Reports from the Italian National Institute of Health (Istituto Superiore di Sanità-ISS) document that most patients with COVID-19 show no or mild symptoms (52% asymptomatic, 17% pauci-symptomatic and 21% with mild symptoms). Ten percent of the patients suffer disease at the severe end of the spectrum, with 3% progressing to critical disease with an overall case fatality rate of approximately 3% (reaching >20% in individuals >80 year old) [1,2]. A more severe SARS-CoV-2 infection has been documented in patients with older age and with coexisting premorbid conditions, such as hypertension, morbid obesity, chronic kidney disease and diabetes [3]. Solid organ transplantation (SOT) recipients may be at increased risk for severe disease and mortality from COVID-19 disease due to immunosuppression and prolonged end-stage organ disease [4]. While some studies have suggested higher morbidity in SOT recipients [5,6,7,8,9,10,11,12,13,14,15], others did not confirm this evidence [15,16,17,18,19,20]. We report here the incidence and outcomes of SARS-CoV-2 infection in a cohort of 291 patients with kidney, pancreas or islet transplant, all of whom received regular follow-ups at the IRCCS Ospedale San Raffaele between February 2020 and April 2021.

## 2. Methods

### 2.1. Study

Recipients aged ≥18 years with a functioning kidney, pancreas or islet transplant, attending the outpatient follow-up clinic at the IRCCS Ospedale San Raffaele between February 2020 and April 2021 were invited to enter this study. The study was completed in April 2021, as from this time on the transplanted subjects began to be vaccinated for SARS CoV-2. None of the included subjects had been vaccinated at the latest available follow-up. Three hundred and two out of 338 (89.3%) accepted to participate and 291 out of 302 (96.3%) completed the study. Most of the patients lived in Northern Italy (*n* = 245, 84.2%), followed by those in Southern (*n* = 29, 10%) and Central Italy (*n* = 17, 5.8%). The study was approved by the Ethics Committee of the Ospedale San Raffaele and all patients signed a written informed consent prior to any study procedure. At the time of the first clinic access during their post-transplant follow up, a comprehensive medical assessment with detailed history and physical examination was performed in all patients. Data on all clinical characteristics, including clinical and pharmacological history, lifestyle factors, comorbidity and body measurements were collected in a structured data collection system. In particular, data on specific symptoms potentially correlated with COVID-19 were obtained asking participants to fill out a questionnaire. A luciferase immunoprecipitation system (LIPS) assay was used to test specific antibodies to different SARS-CoV-2 antigens, as previously described [21]. We tested for IgG antibodies to the virus’ Receptor Binding Domain (RBD) and for IgG antibodies to a second antigenic region, the S2 domain of spike protein to apply a highly specific and sensitive strategy to monitor the SARS-CoV-2 humoral response [22]. After the first study visit, all SOT recipients were recommended to contact the transplant center in case of symptoms compatible with COVID-19 (passive monitoring) and provided periodic information on SARS-CoV-2 infection diagnosis by responding to a phone questionnaire (active monitoring). In case of hospitalization during the study period, clinical data were abstracted from the electronic medical record system of each hospital at which the patient presented. Each case of COVID-19 was defined by a positive reverse transcriptase-polymerase chain reaction (RT-PCR) result for SARS-CoV-2. All COVID-19 RT-PCR positive patients were classified for disease severity according to the WHO severity classification at diagnosis [23]. All COVID-19 RT-PCR positive patients were managed following the consensus promoted by the Italian Society of Organ Transplantation, the Italian Society of Nephrology, the Italian Society of Anesthesia and Intensive Care, and the Italian Group on Antimicrobial Stewardship [24].

### 2.2. Statistical Analysis

Median with inter-quartile range (IQR) was used to present continuous variables, and values were compared using the Mann–Whitney or Kruskal–Wallis test. Categorical variables are reported as frequency or percent and were compared using the Chi-square or Fischer’s exact test, as appropriate. COVID-19 free survival was estimated according to Kaplan–Meier. The time-to-event was calculated from the date of the first locally diagnosed case of COVID-19 in Italy (21 February 2020) to the date of the positive RT-PCR result for SARS-CoV-2 or of the last follow-up visit, whichever occurred first. We studied the association between patient characteristics with time positive RT-PCR result for SARS-CoV-2 using univariate Cox proportional hazards models. Effect estimates, adjusted for age and sex, were reported as hazard ratios (HRs) with the corresponding 95% CI, estimated using the Wald approximation. Associations between baseline variables and COVID-19 severity was assessed by logistic regression. The effect estimates adjusted for age and sex were reported as odd ratios (ORs). Two-tailed *p* values are reported, with *p* value < 0.05 indicating statistical significance. All confidence intervals are two-sided and not adjusted for multiple testing. Statistical analysis was performed with SPSS 24 (SPSS Inc./IBM) and GraphPad Prism version 5.04.

## 3. Results

**Figure 1:**
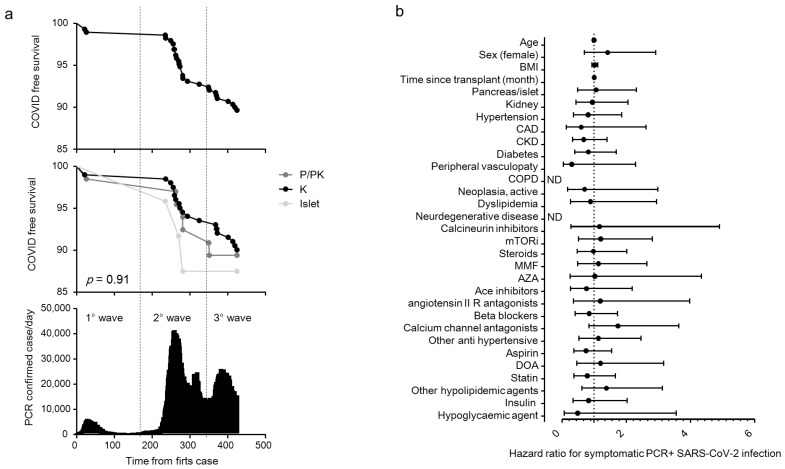
COVID-19 RT-PCR positive subjects in solid organ transplantation (SOT) recipient. Kaplan–Meier COVID-free survival estimates for SOT recipients are in (panel **a**). Survival rate was estimated for all 291 SOT recipients (top panel) or according to the transplant type (kidney (K, n = 201), pancreas (pancreas alone, PA = 10; pancreas-kidney, PK = 56), islet transplant alone (ITA: 24)) (middle panel) in relationship with the development of the COVID-19 pandemic in Italy (bottom panel). The log-rank test was used to test differences in the estimated survival rates among transplant types. The forest plot (panel **b**) shows the hazard ratios (HR) for positive RT-PCR for each factor tested. The univariate Cox regression analysis was adjusted for sex and age. Dots represent the HR, lines represent 95% confidence interval (CI).

**Figure 2:**
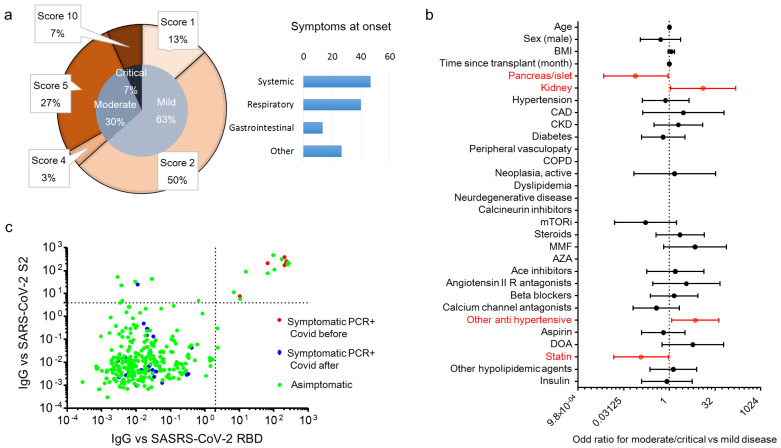
Clinical characteristic and SARS-CoV-2 antibody prevalence in COVID-19 RT-PCR positive subjects in solid organ transplantation (SOT) recipient. WHO severity classification [23] of COVID-19 RT-PCR positive subjects and prevalence of symptoms at diagnosis are in (panel **a**). Of the 291 SOT recipients, 30 (10.3%) tested positive for SARS-CoV-2 RT-PCR during the study period (21 February 2020–24 April 2021). Presenting symptoms were classified as systemic (fever, fatigue/malaise, myalgia/arthralgia), respiratory (cough, dyspnea, sore throat, chest pain), gastrointestinal (diarrhea, vomiting/plasma, abdominal) and others (headache, conjunctivitis, hypo/anosmia, hypo/dysgeusia, skin rash). The forest plot (panel **b**) shows the odd ratios (OR) for moderate/critical disease for each factor tested. The univariate logistic regression analysis was adjusted for sex and age. Dots represent the HR, lines represent 95% confidence interval (CI), and red dots indicate *p* < 0.05. The dot plot (panel **c**) shows the value of IgG antibodies to the virus receptor binding domain (RBD) and the S2 domain of the spike protein. Patients were classified as asymptomatic (green), and symptomatic with a positive SARS-CoV-2 RT-PCR before the antibody test (red) and after the antibody test (blue). Dotted lines indicate the cut-off of antibody test positivity.

We enrolled 290 patients with a kidney (K, *n* = 201), a pancreas (pancreas alone, PA = 10; pancreas-kidney, PK = 56) or islet transplant alone (ITA, *n* = 24) from the cohort of SOT recipients attending a regular follow-up at the IRCCS Ospedale San Raffaele. The baseline characteristics of the entire cohort are summarized in Table 1. The overall median age of the cohort was 56 (47–65) years, and 179 were male (61.5%). Of the 291 SOT recipients, 30 (10.3%) tested positive for SARS-CoV-2 RT-PCR during the study period (21 February 2020 to 24 April 2021, Figure 1a) and their characteristics are reported in Table 2. Most of the cases of positivity occurred during the second and third pandemic waves, while during the first wave the SOT recipients were mainly spared. COVID-19 prevalence was not different among different transplants: 20 out 201 for kidney (10%), 7 out 66 (10.7%) for pancreas ± kidney and 3 out 24 (12.5%) for islet (Figure 1a). The region of residence of SOT recipients did not influence COVID-19 prevalence: 10.6% of patients were form Northern Italy, 10.3% from Southern Italy and 5.9% from Central Italy (*p* = 0.825). There was no statistically significant association between the positivity for SARS-CoV-2 and age, body mass index, time since transplantation, type of transplant, comorbidities and ongoing therapies (Figure 1b) or immunosuppression intensity (triple vs. double/single regimen: HR 1.17 (0.56–2.42). *p* = 0.68). All positive SOT recipients were classified for disease severity following the WHO severity classification at diagnosis: 19 (63.3%) were mild, 9 (30%) were moderate, and 2 were critical and died (Figure 2a) with a crude mortality rate of 6.7%. No patient lost organ function during the SARS-CoV-2 infection. The most common symptoms at presentation were systemic (53.3%: fever, fatigue/malaise, myalgia/arthralgia) and respiratory (40%: cough, dyspnea, sore throat, chest pain). Gastrointestinal symptoms were less prevalent (13.3%: diarrhea, vomiting/plasma, abdominal) as well as other symptoms (26.7%: headache, conjunctivitis, hypo/anosmia, hypo/dysgeusia, skin rash). Kidney transplant (OR 12.9 (1.1–150) *p* = 0.041) and anti-hypertensive therapy other than ACE system blockers, beta blockers and calcium channel antagonists (OR 7.07 (1.18–42.3) *p* = 0.032) were associated with an increased risk to develop moderate/critical disease. Statin therapy (OR 0.116 (0.015–0.926) *p* = 0.042) and pancreas/islet transplant (OR 0.077 (0.007–0.906) *p* = 0.041) were protective against moderate/critical disease (Figure 2b). Immunosuppression intensity was not significantly associated with an increased risk to develop moderate/critical disease (triple vs. double/single regimen: OR 2.25 (0.36–13.9); *p* = 0.38). To estimate the prevalence of asymptomatic disease in our cohort, we analyzed the antibody response of the IgG class to the SARS-CoV-2 Spike protein (both RBD and S2) in all 291 patients at the time of their first study visit (median time after the first case in Italy: 242 days, IQR 201–292). Specific antibody response (positivity for both RBD and S2) was present in 16 out 291 patients (5.5%). Of these, 6 participants were symptomatic and tested positive for SARS-CoV-2 RT-PCR before the study sampling (123 days (IQR 39–220) before), while no history of symptoms and/or positivity for SARS-CoV-2 RT-PCR were reported in the remaining 10 participants (62.5%) (Figure 2c). As expected, all 24 participants who developed symptomatic disease and tested positive for SARS-CoV-2 RT-PCR after the study sampling (96 days (IQR 42–166) after) were negative for the antibody response against the virus. 

## 4. Discussion

The impact of SARS-CoV-2 infection in SOT recipients is an area of intense investigation. Here we present the results of a study in a cohort of SOT recipients undergoing regular follow-up at our institute. Our study generated several interesting findings. First, the COVID-19 cumulative incidence in SOT recipients (10.3%) was slightly higher compared to that of the overall Italian population (6.54%) or age-matched population (age 50–59: 7.4%; age 60–69: 5.9%) during the study period (21 February–24 April 2021; public data from ISS). The modest increase is also evident when compared with the cumulative incidence in Lombardy (7.89% on 24 April 2021), one of the Italian regions most affected by the pandemic and in which 64% of the SOT recipients of our patient population resides. Our data are open to multiple interpretations. The higher cumulative COVID-19 incidence could be to the result of a higher susceptibility to the SARS-CoV-2 infection, to the propensity to refer to healthcare services even in the case of mild symptoms, or to a greater susceptibility to develop a symptomatic form. Second, at the time of the first study visit during post-transplant follow-up we found an overall SARS-CoV-2 antibody prevalence substantially higher than the cumulative incidence of diagnosed SARS-CoV-2 cases. The antibody frequency was approximately 2.6-fold higher than the incidence of SARS-CoV-2 RT-PCR positive SOT recipients, of whom more than half did not report symptoms. As previously reported [25], the specificity of the testing strategy can largely influence the estimated frequency of infection, potentially leading to greatly overestimated population prevalence. For this reason, we applied a two-stage approach, which provided a sensitive and specific approach for detecting SARS-CoV-2 antibodies [22]. Our antibody results underline that testing only patients with symptoms is not effective for identifying all infected SOT recipients. This suggests that the number of SOT recipients infected with SARS-CoV-2 is likely higher than described and that it is difficult to estimate the true impact of the infection in this specific population. Third, we found no association between cumulative incidence and characteristics such as sex, age, body mass index, time since transplantation, type of transplant, comorbidities and ongoing therapies. Fourth, the disease severity in SOT recipients was comparable to that reported in age-matched patients in the general population during the study period (mild disease 68% (asymptomatic: 50% + paucisymptomatic 18%), moderate 23%, severe/critical 9%; public data from ISS), but the estimated crude mortality was higher (age 50–59: 0.6%; age 60–69: 2.7%; public data from ISS). Interestingly, we found no association between disease severity and preexisting comorbidities including hypertension, diabetes, and obesity. Nevertheless, kidney transplant was identified as a risk factor predicting disease severity, while pancreas/islet transplant and statin therapy were protective factors. The fact that COVID-19 severity may be influenced by the transplant type was already suggested. In a recent metanalysis by Raja et al. [26], all-cause mortality in the subset of kidney transplant recipients was 22.0%, vs. 11.8% among liver transplant recipients and 15.6% among heart transplant recipients. In part, this was explained by the high prevalence of acute kidney injury (45.4%) and bacterial urinary tract infection in kidney transplant recipients [26]. Of particular interest, we have, for the first time, reported data about a cohort of islet transplant recipients, who showed a behavior similar to that of whole-organ recipients. Regarding statin therapy, it is a matter of discussion whether its use is associated with a reduced severity or mortality for COVID-19. Conflicting results were published and subgroup analyses suggested the existence of a different susceptibility to statin benefit during SARS-CoV-2 infection [27,28,29]. The suggestion that SOT recipients could benefit from statins is relevant in selecting the target population for future randomized clinical trials. 

Our study has several limitations. First, although we tried to capture all cases of SARS-CoV-2 infection in our transplant cohort, it is possible that patients with very mild symptoms consistent with SARS-CoV-2 did not undergo RT-PCR testing. Second, the fact that we did not observe major differences in disease severity with increasing age or between patients with or without relevant comorbidities, could be due to the relatively small number of cases, narrow age range of recipients and low comorbid heterogeneity observed in our cohort. Third, although all efforts have been made to manage transplant recipients with SARS-CoV-2 infection as recommended by current guidelines, different treatments related to local standards and research protocols from other hospitals may have produced some heterogeneity across pandemic waves [30]. Fourth, the result that renal transplants are at higher risk for moderate-severe COVID-19 when compared to islet or pancreas (w/kidney) transplants is interesting, but the groups had distinct baseline characteristics. In fact, islet and pancreas (w/kidney) recipients had lower BMI, longer interval from transplant, and lower incidence of hypertension and chronic kidney disease. Unfortunately, the number of events in our cohort are not sufficient to perform a multivariate analysis and, even if in our cohort these single variables did not result in a higher risk for moderate/critical disease, we cannot exclude their potential role in determining the result. 

## 5. Conclusions

In conclusion, the incidence of SARS-CoV-2 infection in SOT recipients is likely higher than previously described, as suggested by the presence of viral antibodies, and indicates that estimating the true impact of the infection on clinical outcomes is challenging. In symptomatic recipients, kidney transplant was associated with a higher risk to develop moderate/critical disease, while common risk factors, including age and comorbidities, resulted less relevant for COVID-19 severity. Due to the high estimated crude mortality, symptomatic SOT recipients should be considered at high risk in case of SARS-CoV-2 infection. However, further studies are needed to identify predictive biomarkers and risk factors for disease outcome.

## Figures and Tables

**Table 1:** Baseline characteristics of the entire transplant cohort, and of patients with different type of transplant.

Items	All	Islet	Pancreas ± Kidney	Kidney	*p*
N	291	24	66	201	
Age in years, median (IQR)	56 (47–65)	51 (36–60)	54 (47–59)	57 (49–66)	0.001
Sex M/F	179/112	11/13	41/25	127/74	0.264
Race Caucasian (N (%))	284 (97.6)	24 (100)	64 (97)	196 (97.5)	0.631
Body mass index (kg/m^2^)	24.2 (21.8–26.6)	22.2 (17.8–23.5)	23.2 (20–26.7)	25 (22.5–27)	<0.001
Months since transplant, median (IQR)	53.4 (17–121)	79 (34–131)	75 (24–160)	48 (12–106)	0.005
Comorbidities (N (%))					
Hypertension	227 (78)	11 (45.8)	42 (63.6)	174 (86.6)	<0.001
Coronary artery disease	35 (12)	2 (8.3)	11 (16.7)	22 (10.9)	0.392
Chronic kidney disease	156 (53.6)	2 (8.3)	29 (43.9)	125 (62.2)	<0.001
Diabetes	140 (48.1)	24 (100)	66 (100)	50 (24.9)	<0.001
Peripheral vasculopathy	30 (10.3)	1 (4.2)	7 (10.6)	22 (10.9)	0.585
Chronic obstructive pulmonary disease	1 (0.3)	0 (0)	0 (0)	1 (0.5)	0.799
Neoplasia active	27 (9.3)	1 (4.2)	3 (4.5)	23 (11.4)	0.164
Dyslipidemia	35 (12)	2 (8.3)	5 (7.6)	28 (13.9)	0.327
Neuro degenerative disease	2 (0.7)	1 (4.2)	1 (1.5)	0 (0)	0.043
Baseline therapy					
Calcineurin inhibitor (CNI)	270 (92.8)	21 (87.5)	64 (97)	185 (92)	0.235
Mammalian target of rapamycin inhibitors (mTORi)	58 (19.9)	9 (37.5)	3 (4.5)	46 (22.9)	<0.001
Steroids	133 (45.7)	1 (4.2)	31 (47)	101 (50.2)	<0.001
Mycophenolate mofetil	218 (74.9)	11 (45.8)	59 (89.4)	148 (73.6)	<0.001
Azathioprine	18 (6.2)	6 (25)	4 (6.1)	8 (4)	<0.001
“Intensity” of immunosuppression					
- Triple regimen	122 (41.9)	1 (4.2)	30 (45.5)	91 (45.3)	
○ CNI+antimetabolite+steroid	102 (83.6)	0 (0)	28 (93.3)	74 (81.3)	
○ CNI+mTORi+steroid	14 (11.5)	0 (0)	1 (3.3)	13 (14.3)	
○ mTORi+antimetabolite+steroid	5 (4.1)	0 (0)	1 (3.3)	4 (4.4)	
○ mTORi+CNI+antimetabolite	1 (0.8)	1 (100)	0 (0)	0 (0)	
- Double regimen	162 (55.7)	22 (91.7)	36 (54.5)	104 (51.7)	
○ CNI+antimetabolite	118 (72.8)	13 (59.1)	34 (94.4)	71 (68.3)	
○ CNI+mTORi	28 (17.3)	6 (27.3)	0 (0)	22 (21.2)	
○ CNI+steroid	5 (3.1)	1 (4.5)	1 (2.8)	3 (2.9)	
○ mTORi+steroid	5 (3.1)	0 (0)	0 (0)	5 (4.8)	
○ mTORi+antimetabolite	4 (2.5)	2 (9.1)	1 (2.8)	1 (1)	
○ antimetabolite+steroid	2 (1.2)	0 (0)	0 (0)	2 (1.9)	
- Single regimen	7 (2.4)	1 (4.2)	0 (0)	6 (3)	
○ Antimetabolite	4 (57.1)	1 (100)	0 (0)	3 (50)	
○ CNI	2 (28.6)	0 (0)	0 (0)	2 (33.3)	
○ mTORi	1 (14.3)	0 (0)	0 (0)	1 (16.7)	
Ace inhibitors	51 (17.5)	6 (25)	7 (10.6)	38 (18.9)	0.185
Angiotensin II receptor type 1 antagonists	26 (8.9)	1 (4.2)	5 (7.6)	20 (10)	0.584
Beta blockers	157 (54)	6 (25)	33 (50)	118 (58.7)	0.006
Calcium channel antagonists	125 (43)	3 (12.5)	26 (39.4)	96 (47.8)	0.003
Other anti-hypertensive	106 (36.4)	2 (8.3)	20 (30.3)	84 (41.8)	0.003
Aspirin	185 (63.6)	6 (25)	45 (68.2)	134 (66.7)	<0.001
Direct oral anticoagulant	44 (15.1)	4 (16.7)	12 (18.2)	28 (13.9)	0.688
Statin	134 (46)	7 (29.2)	27 (40.9)	100 (49.8)	0.102
Other hypolipidemic agents	61 (21)	2 (8.3)	8 (12.1)	51 (25.4)	0.02
Insulin	69 (23.7)	18 (75)	19 (28.8)	32 (15.9)	<0.001
Hypoglycemic agent	19 (6.5)	0 (0)	5 (7.6)	14 (7)	0.395

**Table 2:** Characteristics of SOT recipients that tested positive for SARS-CoV-2 RT-PCR during the study period.

Items	SARS-CoV-2 RT-PCR Negative	SARS-Cov-2 RT-PCR Positive	*p*
N	261	30	
Age in years, median (IQR)	56 (47–65)	52 (48–61)	0.341
Sex M/F	163/98	16/14	0.331
Race Caucasian (N (%))	256 (98.1)	28 (93.3)	0.156
Body mass index (kg/m^2^)	24.2 (21.8–26.6)	24 (21.9–26.9)	0.817
Type of transplant			
- Kidney	181 (69.3)	20 (66.7)	0.924
- Pancreas ± kidney	59 (22.6)	7 (23.3)	
- Islets	21 (8)	3 (10)	
Comorbidities (N (%))			
- Hypertension	205 (78.5)	22 (73.3)	0.492
- Coronary artery disease	33 (12.6)	2 (6.7)	0.552
- Chronic kidney disease	143 (54.8)	13 (43.3)	0.251
- Diabetes	127 (48.7)	13 (43.3)	0.7
- Peripheral vasculopathy	29 (11.1)	1 (3.3)	0.337
- Chronic obstructive pulmonary disease	1 (0.4)	0 (0)	1
- Neoplasia active	25 (9.6)	2 (6.7)	1
- Dyslipidemia	32 (12.3)	3 (10)	1
- Neuro degenerative disease	2 (0.8)	0 (0)	1
Baseline therapy			
- Calcineurin inhibitor (CNI)	242 (92.7)	28 (93.3)	1
- Mammalian target of rapamycin inhibitors (mTORi)	51 (19.5)	7 (23.3)	0.631
- Steroids	119 (45.6)	14 (46.7)	1
- Antimetabolites	211 (80.8)	25 (83.3)	1
“Intensity” of immunosuppression			
- Triple regimen	108 (41.4)	14 (46.7)	0.421
- Double regimen	146 (55.9)	16 (50.2)	
- Single regimen	7 (2.7)	0 (0)	

## Data Availability

The data presented in this study are available on request from the corresponding author. The data are not publicly available due to privacy restrictions.

## Abbreviations

K	Kidney
IQR	inter-quartile range
ITA	Islet transplant alone
ISS	Istituto Superiore di Sanità
LIPS	luciferase immunoprecipitation system
OD	Odd ratio
PA	Pancreas alone
PK	Pancreas–kidney
RBD	Receptor binding domain
RT-PCR	reverse transcriptase-polymerase chain reaction
SARS-CoV-2	severe acute respiratory syndrome coronavirus 2
SOT	Solid organ transplantation
